# LBD around the world – The Latin American experience

**DOI:** 10.1002/alz.091443

**Published:** 2025-01-09

**Authors:** Miguel Germán Borda, Salomón Salazar‐Londoño

**Affiliations:** ^1^ Stavanger University Hospital, Stavanger, Rogaland Norway; ^2^ Pontifical Xaverian University, Bogota Colombia

## Abstract

Up to date, there are no precise reports of the prevalence of dementia with Lewy bodies (DLB) in Latin America. This can be explained by the lack of research studies and general little awareness about the disease. Notably, collaborative clinical studies are lacking, and DLB patients remain underrepresented despite their significant morbidity.

The few studies investigating DLB in Latin America have been based on small samples, resulting in disparities across the region. Challenges include variability in follow‐up regimens and protocols, prompting the need for harmonization to assess the practical benefits for patients. Future efforts should prioritize collaborative initiatives and address knowledge gaps to enhance the understanding and management of DLB in Latin America.

Here, we describe the first multi‐center initiative for the study of DLB in Colombia.

The main objectives of this initiative are: 1) to identify the level of DLB awareness in memory clinics in Colombia, 2) to analyze the use of clinical instruments and biomarkers for DLB, 3) to characterize the Colombian DLB population, and 4) to explore the potential usefulness of biomarkers in combination with other clinical features to improve diagnostic accuracy and prognostic prediction, especially during the early stages of the disease.

This initiative is linked to the European DLB consortium (*E*‐DLB) using harmonized procedures and protocols, allowing the opportunity to directly compare clinical and biomarker features across continents.

